# Cut-off value of *D. pteronyssinus* specific IgE in double negative patients Der p 1 and Der p 2 and its clinical repercussion

**DOI:** 10.1038/s41598-021-03005-4

**Published:** 2021-12-08

**Authors:** Antonio Letrán, Ignacio García, Marisa Espinazo-Romeu, Carmen Moreno-Aguilar, Francisco Moreno

**Affiliations:** 1 Servicio de Alergología e Inmunología, CM ASISA Lobatón, Cádiz, Spain; 2 University of Cordoba, Cordoba, Spain; 3 GC01 Laboratorio de Inmunología y Alergia, Maimonides Biomedical Research Institute of Cordoba (IMIBIC)/Reina Sofia University Hospital/University of Cordoba, Avenida Menéndez Pidal, S/N, 14004 Córdoba, Spain; 4 National Network ARADyAL, Health Institute Carlos III, Madrid, Spain; 5 Department of Immunology and Allergy, Reina Sofia University Hospital, Cordoba, Spain

**Keywords:** Immunology, Diseases

## Abstract

Accessibility to more precise diagnostic techniques such as component resolved diagnostics (CRD), provides us with an important advance in diagnostic aspects as well as treatment. The subject of this study aims to better understand the profiles of sensitization to Der p 1, Der p 2 and Der p 23 and to know to what extent their use could help us in optimizing the decision-making for their treatment with Specific Immunotherapy. Cross-sectional study of subjects older than 5 years, diagnosed with allergy to HDM using skin prick test and sIgE, with symptoms of rhinitis and/or asthma. Total and specific IgE was determined to *D. pteronyssinus*, nDer p 1, rDer p 2 and rDer p 23 using ImmunoCAP. 240 patients were recruited (97.1% rhinitis and 46.25% rhinitis and asthma). Four different phenotypes were observed: positive or negative for sIgE nDer p 1 and/or IgE rDer p 2. 17% of these patients sIgE were double negative for Der p 1 and Der p 2 (increasing with age and with significantly lower sIgE levels than the rest of the groups). Using ROC curves, value less than 2.18 KU_A_/L for *D. pteronyssinus* sIgE gave us a sensitivity and specificity of 0.882 and 0.985, respectively, to double negative IgE nDer p 1 and IgE rDer p 2 group. Despite positive SPT and sIgE to *D. pteronyssinus*, 17% of the studied population is IgE nDer p 1 and IgE rDer p 2 double negative, with a cut-off value of 2.18 KU/L, which is very relevant for taking of decisions in prescription of AIT. The double positive population sIgE nDer p 1 and IgE rDer p 2 is associated with asthma compared to the other groups and this does not seem to be influenced by IgE rDer p 23.

## Introduction

House dust mite (HDM) represents a major source of allergens affecting a high number of people worldwide^1^. In moderate climate countries, mite allergic patients, are co-exposed and co-sensitized to allergens from both *D. pteronyssinus (Dptr)* and *D. farinae* species^2^. Batard et al. (2016) suggested that to design allergen products suitable to treat HDM—allergic patients in various parts of the world, it is very important to understand the IgE sensitization profile to allergens from *Dermatophagoides* species. Among mite group 1 and 2 allergens are clearly the most important ones, both in terms of prevalence and relative contribution to total specific IgE (sIgE) reactivity against HDM allergens, being the most relevant to European patients^3^. Molecular allergy diagnostics has become an important tool in daily routine in allergy centers. The knowledge about major and minor allergen components and their prevalence opened new possibilities in allergy diagnostics and therapy^4,5^. As a consequence of these findings, molecular diagnostics for major allergen components should be mandatory before immunotherapy (ITA) in HDM allergic patients. For diagnostic purposes, measurement of specific antibodies to HDM extracts could be a safe and routine procedure with high value estimates of sensitivity and specificity in both nDer p 1 (Derp1) and rDer p 2 (Derp2) supported the use in the diagnosis of *Dptr* and optimizing immunotherapy outcome^5^. Previous studies have shown that sensitization HDM patients’ profile is related with the exposition to HDM^4,6,7^. But the current diagnosis of *Dptr* allergy is based on a clinical history of allergic symptoms and a Skin-Prick Test (SPT) by using crude extracts from mites, without sufficient knowledge of Der p 1 and Der p 2 profile in a population exposed to a high allergenic load for HDM^8^ or how to use it in daily clinical practice. On the other hand, Der p 23 was identified as a major *D. pteronyssinus* allergen. Der p 23 is a protein with a molecular weight of 11 KDa. Pang et al.^9^, showed that Der p 23 was recognized in > 70% of HDM-allergic patients, and the Der p 23-sIgE levels of the patients were comparable to the two major HDM allergens, Der p 1 and Der p 2^9^. The aim of the present study was to investigate the profile of Derp1, Derp2 and Der p 23 (Derp23) allergens in mite-sensitized patients and draw practical conclusions for daily practice or involvement in making decisions for immunotherapy.

## Materials and methods

### Study population

We conducted a cross-sectional study covering 240 consecutive HDM allergic patients attended in Southern Spain (Clínica Lobatón and QuirónSalud Gibraltar area, both in the coastal area). The local weather is warm and humid, and mites are the most relevant inhalant allergens^8^. Routine diagnosis was established through the association between a typical history of allergic symptoms for either rhinitis, according to ARIA criteria^1^ or asthma according to GEMA criteria^10^ and the presence of sensitization (positive Skin Prick Test (SPT), all of them with the intention of being treated with AIT. This study was approved by the Clinica Lobatón, S.L.P. Ethic Committee and written informed consent from all patients was obtained.

## Main determinations

### Skin prick testing

SPT was performed with a solution for HDM from LETI lab. SLU. The procedure agreed with published guidelines^11^. A positive response was defined as the presence of a wheal with a diameter ≥ 3 mm. Histamine chloride 10 mg/mL was used as a positive control.

### Serological analysis

Total IgE levels, sIgE to *Dptr*, Derp1, Derp2, Derp23, were measured by ImmunoCAP (ThermoFisher Scientific, Uppsala, Sweden) according to the manufacturer’s instructions. Total IgE levels were expressed in international units per unit volume (kU/L), sIgE levels were expressed in kU_A/_L. Values ≥ 0.35 kUA/L were considered positive.

### Statistical analysis

Demographic characteristics of patients were expressed as mean ± standard deviation (SD) for continuous variables and as frequency distribution for categorical variables. All the cellular variables were expressed as median (minimum, maximum).

Wilcoxon rank sum, Kruskal–Wallis chi-squared and Fisher test with alternative hypothesis of two-sided tests were used to determine the overall differences between the four groups and clinical symptoms: sIgE nDer p1 (IgEDerp1) and IgE rDer p2 (IgEDerp2) positive patients (DP), IgEDerp1 positive and IgEDerp2 negative patients (DP1), IgEDerp1 negative and IgEDerp2 positive patients (DP2) and IgEDerp1 and IgEDerp2 negative patients (Double Negative) (DN). Furthermore, post-hoc tests were performed for multiple comparisons using None, Holm and Bonferroni correction of p-values. Samples were significantly different when p-value < 0.05 in the three methods. ROC curves were performed under parametric conditions. R statistical software (versión 3.5.0; The R Foundation, Vienna, Austria) was used to perform all the analyses.

### Ethics

The study was approved by the local Ethical Committee of our Institution with the recommendations of the Declaration of Helsinki. Informed consent was signed by all subjects or parents/guardians for participants < 18 years old.

## Results

Two hundred and forty subjects were recruited for this study. All of them was sensitized to *Dptr* by SPT and IgE to *Dptr* by Immunocap System. Characteristics of the study population are summarized in Tables 1, 2 and 3.

**Table 1:** Demographic, clinical and serological characteristics of the studied population. Demographic characteristics of studied population. Data are represented as: Mean ± S.D.; Median (Max–min) and variance valor.

Patient’s characteristics				
Sex (Male/Female)	146/94			
Age (year) Mean ± S.D Median (max–min) Variance	33.12 ± 13.33 36 (62–6) 177.67			
	DN	DP1	DP2	DP
	37.78 ± 15.55 40 (62–6) 241.93	30.71 ± 13.41 31 (52–7) 179.72	26.24 ± 14.37 20 (56–7) 206.49	33.08 ± 12.06 35 (62–12) 145.52

**Table 2:** Rhinitis and asthma characteristics depending of the group studied. Data are represented: n = number of patients; % percentage. GRADE corresponding to ARIA or GEMA classification.

Clinical characteristic				
Groups	DN	DP1	DP2	DP
RHINITIS (n, %) No rhinitis Grade 1 Grade 2 Grade 3	Total patients: 233 (97.1%) 7 (2.9%) 23 (9.6%) 109 (45.4%) 101 (42.1%)			
	2 (4.9%) 3 (7.3%) 26 (63.4%) 10 (24.4%)	0 (0%) 4 (23.5%) 5 (29.4%) 8 (47.1%)	3 (14.3%) 3 (14.3%) 8 (38.1%) 7 (33.3%)	2 (1.2%) 13 (8.1%) 70 (43.5%) 76 (47.2%)
Asthma (n, %) No asthma Grade 1 Grade 2 Grade 3	Total patients: 111 (46.25%) 129 (53.75%) 51 (21.25%) 37 (15.42%) 23 (9.58%)			
	29 (70.73%) 5 (12.19%) 6 (14.63%) 1 (2.44%)	9 (52.94%) 7 (41.18%) 1 (5.88%) 0 (0%)	14 (66.67%) 3 (14.28%) 1 (4.76%) 3 (14.28%)	77 (47.83%) 36 (22.36%) 29 (18.01%) 19 (11.80%)

**Table 3:** IgE value of studied population. Data are represented as: Mean ± S.D.; Median (max–min) and variance valor. D1: complete extract of *D. pteronyssinus*.

IgE valor (KU/L)				
Groups	DN	DP1	DP2	DP
Total IgE: Mean ± S.D Median (max–min) Variance	289.54 ± 377.63 146.75 (2000–2.27) 142,604.93			
	274,65 ± 532,71 86 (1855.0–2.27) 283,782.41	241.69 ± 473.42 65.95 (1779.0–4.1) 224,124.58	351.06 ± 469.01 171.65 (2000–17.5) 219,966.50	268.67 ± 286.93 172 (2000.0–7.80) 82,326.41
sIgE D1: Mean ± S.D Median (max–min) Variance	29.79 ± 34.57 13.90 (181.00–0.07) 1195.36			
	3.00 ± 7,68 0,89 (41.00–0.07) 59.02	13.18 ± 19.33 6.66 (77.5–0.39) 124.09	21.32 ± 32.55 11.60 (100–0.68) 1059.26	35.75 ± 34.72 23.20 (181–1.22) 1205.49
sIgE Der p 1: Mean ± S.D Median (max–min) Variance	13.96 ± 21.35 4.33 (100–0.00) 455.66			
	0.09 ± 0.05 0.1 (0.28–0.0) 2.24 × 10^−34^	9.69 ± 11.14 4.76 (35.4–0.39) 124.09	0.07 ± 0.05 0.1 (0.14–0.00) 0.00	17.08 ± 21.17 8.45 (100.00–0.47) 448.25
sIgE Der p 2: Mean ± S.D Median (max–min) Variance	16.61 ± 22.79 6.58 (100–0.00) 519.42			
	0.09 ± 0.05 0.10 (0.31–0.00) 0.00	0.08 ± 0.08 0.10 (0.29–0.00) 0.01	17.60 ± 24.16 9.21 (100.0–0.40) 583.86	20.10 ± 22.72 11.20 (100.0–0.38) 516.35
sIgE Der p 23: Mean ± S.D Median (max–min) Variance	4.95 ± 12.79 2.13 (95.90–0.00) 163.47			
	0.89 ± 2.01 0.08 (7.60–0.0) 4.06	1.33 ± 2.25 0.17 (5.70–0.04) 5.04	3.74 ± 3.07 5.43 (5.59–0.19) 9.44	6.30 ± 15.53 2.80 (95.90–0.00) 241.16

Prevalence of sensitization and levels of sIgE to *Dptr* and their components nDer p 1, rDer p 2 and rDer p 23.

**Figure 1:**
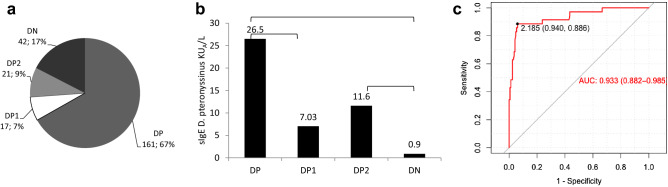
Prevalence of sensitization and levels of IgE in the different phenotype studied. (**a**) Prevalence of sIgE reactivity to rDer p 1, rDer p 2 in HDM allergic patients in southern o Spain (n = 240). (**b**) Mean value of sIgE D. pteronyssinus to each phenotype. Bar shows statistical difference between phenotype. P-value < 0.05. (**c**) ROC curve about total IgE *D. pteronysssinus.* Cut-off point from which the complete extract-specific IgE valor is positive, but IgE nDer p 1 and IgE rDer p 2 will be negative.

67% of the patients were found sensitized Double Positive (DP), 17% were not sensitized to nDerp1 and rDerp2 (DN) and IgEDerp1 positive and IgEDerp2 negative patients (DP1) and IgEDerp1 negative and IgEDerp2 positive patients (DP2) were found sensitized in a 7% and 9% respectively, being DN significantly higher than the proportion of DP1 or DP2 patients (Fig. 1a). However, the production of sIgE against *Dptr* varies significantly between DP1 and the rest of the groups, being significantly lower in DN group, being the range of median valor of 26.5 KU_A_/L and 0.90 KU_A_/L, respectively (Fig. 1b). ROC curve analysis show that a cut-off point to complete extract-specific IgE where values lower than 2.18 KU_A_/L implies that sIgE nDerp 1 and sIgE rDerp 2 was negative (Fig. 1c).

**Figure 2:**
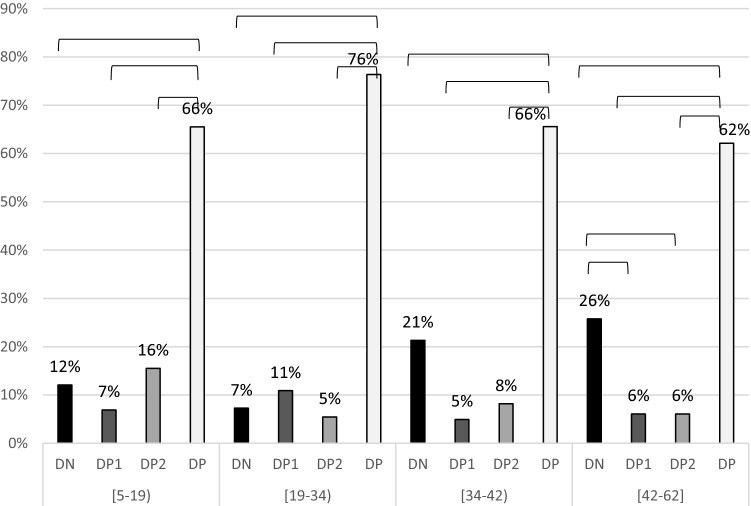
Distribution of the different phenotype in function of the age. Bar means statistical difference p-value < 0.05.

Adjusted the sIgE production to *Dptr* by each group (DP, DP1, DP2 and DN) according to age quartiles, there is an increasing trend of the DN group according to age, as well as a slight decrease in group DP2 (Fig. 2).

**Figure 3:**
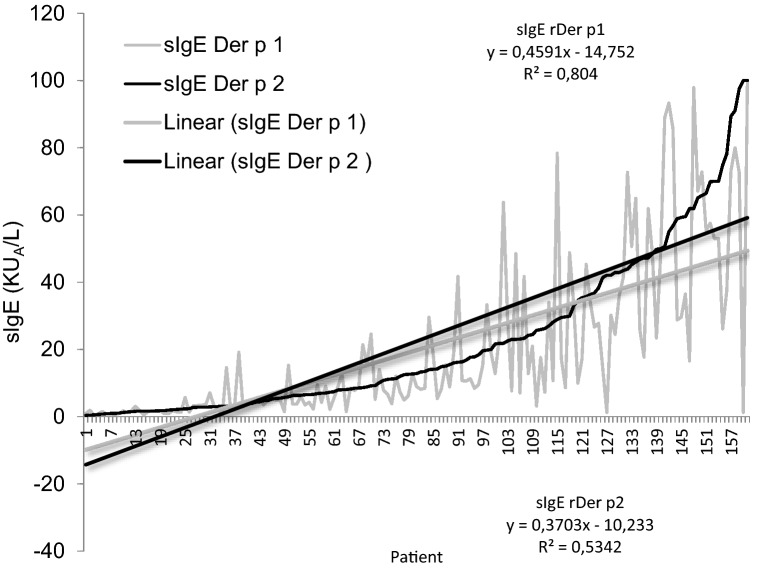
Graphic representation of sIgE nDer p1 and sIgE rDerp2 value for each patient. In the graphic is represented lineal correlation and R^2^ valor. Correlation Pearson = 0.53567; Correlation Spearman = 0.54864.

The production of IgEDerp1 versus IgEDerp2 does not maintain a linear correlation, with statistically significant curve slopes (Fig. 3).

Relationship between sIgE nDer p 1, sIgE rDer p 2 and sIgE rDer p 23 in each group.

**Figure 4:**
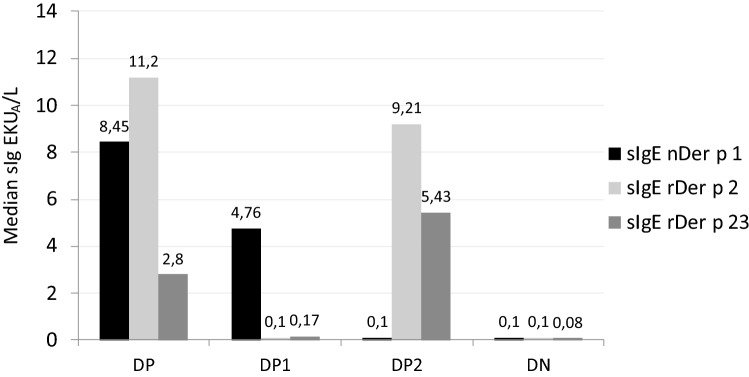
sIgE median value to different components of *D. pteronyssinus* for each phenotype studied.

In the total sample, in comparison DP, DP1 and DP2 groups was observed as DP had higher levels for IgEDer p1 and IgEDerp2 between these groups without reaching statistical significance between them (Fig. 4). sIgE rDer p 23 (IgEDerp23) is present in 69.2% of the total sample, with a clear difference in each group, both in frequency and in levels of sIgE. IgEDerp23 positive in DP group positive was present in 90% of the sample. In DP1 and DP2 was present in 50% of the sample and in the DN group was present in 75% of the sample IgEDerp23 showed significant values in DP and DP2 groups (median 2.99 and 5.43 KU/L sIgE respectively), with respect to DP1 or DN. In DN group IgEDerp23 showed a high frequency of low values of IgEDerp23 that constitute 75% (mean sIgE 0.84 ± 1.9).

Prevalence of sensitization and levels of sIgE to nDer p 1, rDer p 2 and rDer p 23, according to clinical diagnosis, in each group.

### Rhinitis

**Figure 5:**
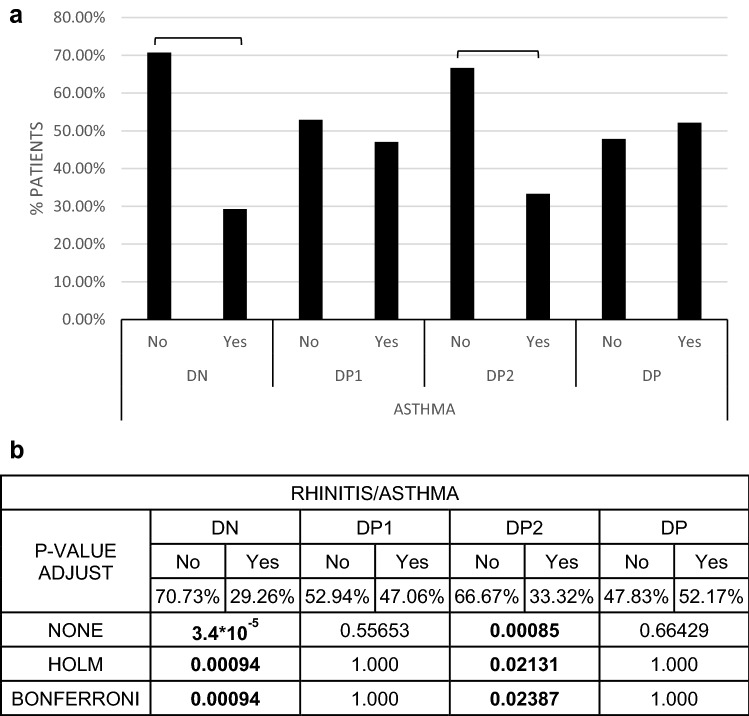
Rhinitis/asthma distribution for each phenotype. (**a**) Percentage of patient in each group suffering rhinitis (no)/rhinitis + asthma (Yes). (**b**) p-value using different statistical adjust model. When the p-value is lower than 0.05 in the three type of adjust, the sample are considered statistically difference.

There were significant differences between the studied groups in relationship with presence of rhinitis, being predominant in the whole sample. The percentage of patients where rhinitis predominated versus asthma was significantly higher in the DN and DP2 group (Fig. 5). In the DN group, persistent mild rhinitis (grade 2) predominated. The rest of the groups severity grades 2 and 3 predominated over grade 1 (Fig. 1 suppl.). We did not find statistically significant differences in the production of IgEDerp1, IgEDerp2 or IgEDerp23 in the different degrees of severity.

### Asthma

There was a different distribution of asthma severity depending on the groups. In the DP group, the three degrees of severity of asthma coexisted, with no predominance of one over the other. Grade 1 and 3 predominated in the DP2 group. Grade 1 clearly predominated in the DP1 group with grade 3 absent. Grade 1 and 2 predominated in the DN group. There were no differences in the IgEDerp23 values in the absence of asthma or its presence in any of the degrees of severity (Fig. 2 suppl.).

As the same as rhinitis, comparing the different groups with each other, DP, followed by DP2 and DP1 group, showed highest to lowest value of sIgE, without significant differences. There were statistically significant differences between the DP group with respect to DN (Fig. 5).

## Discussion

In the studied population, patients with ≥ 5 years old have been selected. Skin Prick Test positivity to *Dermatophagoides pteronyssinus* was a main criterion to include the patients in this study together to a medical record with symptoms concordant with dust house mite allergy. Although, it is necessary a standardization of the allergenic extracts used in diagnosis, like is showed by Brunetto et al.^12^ and Casset et al.^13^, we have taken the same HDM extract used in the article published by Jiménez-Feijoo et al.^14^, where the authors showed that all patients sensitized exclusively to rDer p 23 had a positive SPT to *D. pteronyssinus*, so we can consider that the extract used is useful in our population. Inclusion criteria in the different groups were sensitization profile defined at least for IgEDerp1, IgEDerp2 or IgEDerp23, as well as their sIgE to *Dptr* must be positive ≥ 0.35 kUA/L measured by Immunocap System^15^. The allergenic burden for house mites is significantly higher in southern European countries^16^. However, for the diagnosis of sensitization to HDM, IgEDerp1 and IgEDerp2 are sufficient as the most relevant allergens and capable of diagnosing > 95% of patients in Europe and Chines^3,17^. While other studies showed a lower IgE responses to Der p 1 and Der p 2 for Australia (74–77%) and Singapore (63%)^18^. Our series shows the presence of the 4 phenotypes in production for IgEDerp1 and IgEDerp2^3,6,7,19,20^. The majority group DP (IgEDerp1 and IgEDerp2 double positives), significantly higher, followed by DN (double negative to IgEDerp1 and IgEDerp2) being the second most relevant group. Groups DP1 and DP2 formed a minority group. DN group constitutes 17% in the total sample. However, we can see that the percentage of DN patients varies according to age, with an increasing trend towards older age. This indicates more years of exposure increases the possibility of developing sensitization to minor allergens present in mites. Although the pattern of sensitization is defined at relatively early ages^15^, everything seems to indicate that there is an important group that will progressively increase from 12% (in those under 18 years of age) to 26% (in those over 45 years of age). DN group is a very significant value to consider in two fundamental aspects: initiation of AIT or inclusion in clinical trials for this treatment. This double negative group to IgEDerp1 and IgEDerp2 is sensitized to minority allergens^21^. DN group patient’s shows a low response in the production of sIgE for the complete extract of *Dptr*, therefore it may influence its use as an AIT response biomarker^22^. Although the use of AIT in those that are only IgEDerp1 or IgEDerp2 positive is justified, since the recommendation is that the extracts contain known amounts of these allergens, the use of AIT in the double negatives for IgEDerp1 and IgEDerp2 is empiricism, we do not know the answer. In daily clinical practice, we cannot know which minority allergen the patient is allergic to and whether the extract administered contains it, or whether it is equally present in all its batches, or its concentration. Unless for immunotherapy clinical trials, this double negative group Derp1 and Derp2, should be excluded.

In daily clinical practice, a clinical history of allergy to HDM, with concordant positive skin tests, let us diagnosis to the patients. Despite the progress and development evolution makes by the components resolving diagnostic (CRD), these are not routinely performed for diagnosis, either because of the cost or because they are not considered useful enough. With the results obtained, values of sIgE to complete extract of *Dptr* in population older than 5 years old, lower than 2.18 KUA/L; we would be possibly in the double negative group for IgEDerp1 and IgEDerp2. The cost for testing IgEDerp1 and IgEDerp2 prior to AIT could be reduced by selecting only patients with lower values at 2.18 KU_A_/L. Logically, this is valid only in the geographical area with a similar allergenic load and more studies are needed to establish an overall value that discriminates the DN group.

Our results show that the levels of IgEDerp2 is higher than IgEDerp1^3,6,7,19,20^ without reaching statistical significance. However, since the sIgE production describes a non-linear curve and the commonly used method is an ELISA whose curve is not linear either, we can only say that there is a higher sIgE production for HDM group 2. This cannot be used to measure the quantity of allergens present in the immunotherapy extracts. But both Derp1 and Derp2 allergens must be present, at least in the same proportion.

IgEDerp23 is present in a very different grade in each of the groups studied, which for the total sample is 63%. IgEDerp23 seems to be a relevant allergen both in frequency and in sIgE production in the USA, but it does not seem to be as relevant in Europe^3^, Although, its relevant is increasing in the last years and is in relationship with the geographic distribution and climate conditions^18,23^. In our sample, IgEDerp23 is mainly present in the double positive group IgEDerp1 and IgEDerp2 along. It is very striking, in the group positive for IgEDerp1 it does not have levels of IgEDerp23. Both are allergens present in the faeces. However, if it is present in the positives only to IgEDerp2 group, an allergen present in the body of the mite. It appears that in the absence of Derp1 sensitization, the immune system responds to Derp23 as an allergen present in faeces. Patients sensitized only to Derp1 or only Derp2, both constitute a small number of the sample (16%)^14,21^. In our results, in the group double negative to IgEDerp1 and IgEDerp2 (DN), the 75% of them are IgEDerp23 positive, with low values of sIgE with mean 0.89 ± 2.01 KU_A_/L and median 0.08 (7.60–0.0) KU_A_/L. Everything seems to indicate that IgEDerp23 has a high prevalence but little relevance, since in the DP the most frequent is sensitized to Derp1, Derp2 and other allergens including Derp23, while in the DN group, the production of sIgE produced is very low and contributes little to the total sIgE compared to IgEDerp23 produced. In a recent study in children, where the pattern of sensitization Derp1, Derp2 and Derp23 was studied^14^, the DN percentage was 9.4%, associating the presence of moderate–severe asthma when the three allergens coexisted. However, in the DN rhinitis prevailed over asthma. In a recent study, no association was found between the presence of IgEDerp23 and response to AIT, although it was present in 88% of the sample studied^24^.

There is great controversy whether the frequency or levels of IgEDerp1 or IgEDerp2 or other CRDs, is associated with the respiratory symptoms of patients, be it rhinitis or asthma. As Posa describes in his work^15^, summarized with very interesting conclusions in a review^25^, higher exposure to mites were associated with a broader polymolecular IgE sensitization pattern. Participants achieve the broadest IgE sensitization stage, depending on the sensitization pattern A, B or C. The ABC pattern had significantly higher risk of mite-related AR and asthma than unsensitized participants. IgEDerp1 or IgEDerp23 at age 5 years or less predicted asthma at school age. Five hundred and one adolescent were recruited in Salzburg, showing that in alpine areas, less exposure to allergens, less sIgE response and less clinical severity were found, but not less house dust mite allergic people. Group 2 allergens predominated in the houses and the response of sIgE production (measured by ISAC micro-array platform) was greater for IgEDerp2^26^. With our results we have not found differences between rhinitis and asthma in the total sIgE versus complete extract, no differences for the group IgEDerp1 and/or IgEDerp2, but if it appears in the double negative group IgEDerp1 and IgEDerp2 where rhinitis predominates. This is very curious, because in the group IgEDerp1 negative but IgEDerp2 positive that also has IgEDerp23, there is no higher frequency of asthma, this makes us think that by itself both IgEDerp2 and IgEDerp23, are not relevant to the presence of asthma.

Fifty-seven of the studied patients were younger than 18 years old, corresponding to 23.6% of our studied population. 64.9% of them belong to DP group, 15.8% to DP1, 12.28% to DN and 7.01% of DP2 group. Of this patients group, a 57% suffered asthma, with this distribution: in the DP group the 67% of them suffered asthma, being 48% moderate asthma and 52% intermittent asthma; in the DP1 group, the 33% of them suffered asthma being 66.7% moderate and 33.3% intermittent asthma; in the DP2 a 50% suffered asthma intermittent and in the group of DN the 42% of these patients suffered moderate asthma. These data are in agreement with data published by Jiménez-Feijoo^14^.

ROC analysis was performed to evaluate the ability of IgE antibodies (IgE Abs) to crude *Dptr* and pure *Dptr* components to predict immediate asthmatic response (IAR) to bronchoprovocation^27^. The AUCs (Area under curve) for the levels of IgE Abs to IgEDerp1 and IgEDerp2 were 0.913 (95% confidence interval (CI), 0.817e1.000) and 0.906 (95% CI 0.801e1.000), respectively, comparable for *Dptr* (0.920; 95% CI 0.828e1.000). It shown the relationship between the levels of IgE Abs and IAR positivity predicted using a logistic regression model. The curves show ascending slopes in which an increasing IgE concentration value corresponds to a higher probability of IAR positivity. It shown diagnostic values of IgE Abs to crude *Dptr*, IgEDerp1, and IgEDerp2 at different cut-off points. IgE Abs to crude *Dptr* showed good diagnostic values at the cut-off point of 3.5 kUA/L. Taking these data together with our results, the patients included in the DN group (IgEDerp1 and IgEDerp2 double negative) with asthma and lower values of 3.5 KU_A_/L to HDM, we must think of another allergen or cause as responsible for your asthma.

The differences in the response or pattern of sensitization to the different allergens and the presence or absence of asthma are documented, as well as their possible relationship with age^28–30^. Symptomatic patients recognize more HDM allergens than asymptomatic subjects. The frequency of sensitization to Derp1 and Derp2 was the same, although not all positive patients were sensitized to both allergens. Sensitization to Derp5 was also highly prevalent in both groups. Differences were observed in the frequency of sensitization to Der p 7 and Derp23. The sIgE to Der p 4, Der p 10, Der p 11, Der p 14, Der p 15, Der p 18 and Der p 21 were no significant difference between the symptomatic patients and asymptomatic subjects^29^, low sample size (n = 17 and 19 symptomatic and asymptomatic, respectively). Significantly higher concentrations of IgEDerp1 and IgEDerp2 were observed in patients with asthma than in those without asthma^23,31^. As same as our results, 11% of the patients were DN and sIgE levels to certain HDM allergens Derp1, Derp2, Der p 5 and Derp23 were significantly higher in asthmatic children than children without asthma. There was also no correlation between age and the number of recognized allergens. Patients with IgE reactivity to Der p 5 and a probability of 85% of having asthma^30^.

As Vidal et al.^31^, in the DP group there is a greater presence of asthma than in the DN in a significant way, but we also found it in the DP2 group (IgEDerp2 without IgEDerp1). However, we find differences regarding the severity of asthma, being predominantly milder (grade 1) for the DP1 and DN group with respect to DP or DP2. The frequency of asthma does not vary by groups or the presence or absence of Derp23.

Anyway, we have to understand that, the differences in the production of sIgE for rhinitis and asthma in allergic patients allergic to HDM, is artificial.

## Conclusion

The availability of CRD allows us to visualize the sensitization profile of patients allergic to complete allergens. Possibly the same phenomenon is being viewed from different perspectives. In this sense, the debate between prevalence and relevance arises. Even considering only the most prevalent for HDM, available in daily practice, such as Derp1, Derp2 and Derp23, we found serious differences in various studies. In our study we found a clinical relationship, in rhinitis with or without asthma, regarding the groups double positive to IgEDerp1 and IgEDerp2, positive only to one of them or double negative to both. In addition to being able to establish a point of cut-off from which follows the complete extract, allows us to predict the double negative group IgEDerp1 and IgEDerp2 and its clinical repercussion. Derp23 is a prevalent allergen but we found no clinical relevance. More robust studies are needed in the number of patients and in representative areas of high HDM allergenic load, so that the CRD tools can be used as a diagnosis, or prognosis or marker of some clinical characteristic useful in daily practice, for example indication of AIT.

## Supplementary Information

Supplementary Figures.
