# Supplementary material for: Cut-off value of D. pteronyssinus specific IgE in double negative patients Der p 1 and Der p 2 and its clinical repercussion
Source: Sci Rep. 2021 Dec 8;11:23585. doi: 10.1038/s41598-021-03005-4 (PMC8655041; doi:10.1038/s41598-021-03005-4)
Supplement: Supplementary file 1 — Supplementary Figures. [file 41598_2021_3005_MOESM1_ESM.pdf]

a)

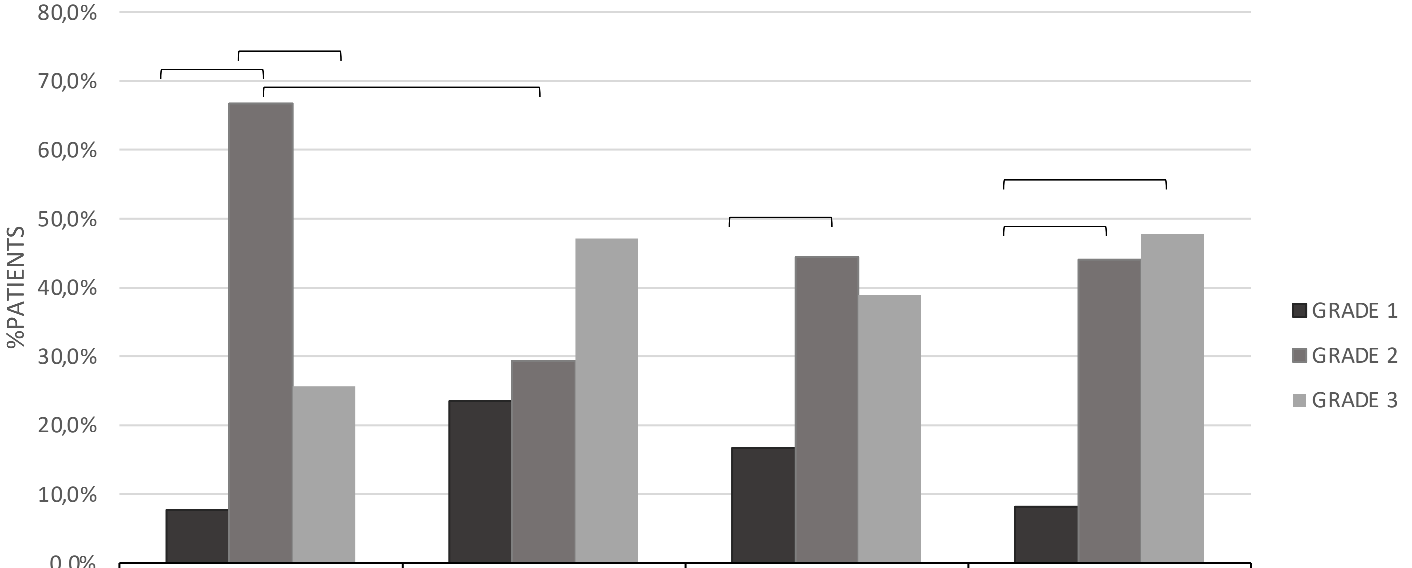

	DN	DP1	DP2	DP
GRADE 1	7,7%	23,5%	16,7%	8,2%
GRADE 2	66,7%	29,4%	44,4%	44,0%
GRADE 3	25,6%	47,1%	38,9%	47,8%

b)

	RHINITIS						
	P-VALUE ADJUST	DN-DP1	DN-DP2	DN-DP	DP1-DP2	DP1-DP	DP2-DP
GRADE 1	NONE	0.00467	0.06846	0.90021	0.28350	0.00658	0.08849
	HOLM	0.18698	1.000	1.000	1.000	0.24999	1.000
	BONFERRONI	0.77109	1.000	1.000	1.000	0.43419	1.000
GRADE 2	NONE	**0.00014**	0.03437	0.03097	0.08080	0.08836	0.96607
	HOLM	**0.00706**	0.99681	0.92899	1.000	1.000	1.000
	BONFERRONI	**0.00936**	1.000	1.000	1.000	1.000	1.000
GRADE 3	NONE	0.01168	0.09771	0.00956	0.37657	0.94272	0.33916
	HOLM	0.39723	1.000	0.34429	1.000	1.000	1.000
	BONFERRONI	0.77109	1.000	0.63119	1.000	1.000	1.000

	RHINITIS				
	P-VALUE ADJUST	DN	DP1	DP2	DP
G1-2	NONE	**$7.9*10^{-12}$**	0.41725	**0.00039**	**$7.2*10^{-7}$**
	HOLM	**$5.2*10^{-10}$**	1.000	**0.01854**	**$4.0*10^{-5}$**
	BONFERRONI	**$5.2*10^{-10}$**	1.000	**0.02604**	**$4.8*10^{-5}$**
G1-3	NONE	0.00192	0.00497	0.00291	**$1.2*10^{-7}$**
	HOLM	0.08460	0.19398	0.12215	**$7.4*10^{-6}$**
	BONFERRONI	0.12690	0.32827	0.19196	**$8*10^{-6}$**
G2-3	NONE	**$1.9*10^{-5}$**	0.04300	0.54676	0.69166
	HOLM	**0.00098**	1.000	1.000	1.000
	BONFERRONI	**0.00124**	1.000	1.000	1.000

Fig. 1 suppl.. Rhinitis grade distribution for each phenotype considering ARIA classification. a) Percentage of patient in each group suffering rhinitis. Bar mean statistical difference. $P<0.05$. b) p-value using different statistical adjust model. When the p-value is lower than 0.05 in the three adjust the sample are considered statistically difference. G1-2,G1-3, G2-3, mean de relationship between both different grade of severity.

a)

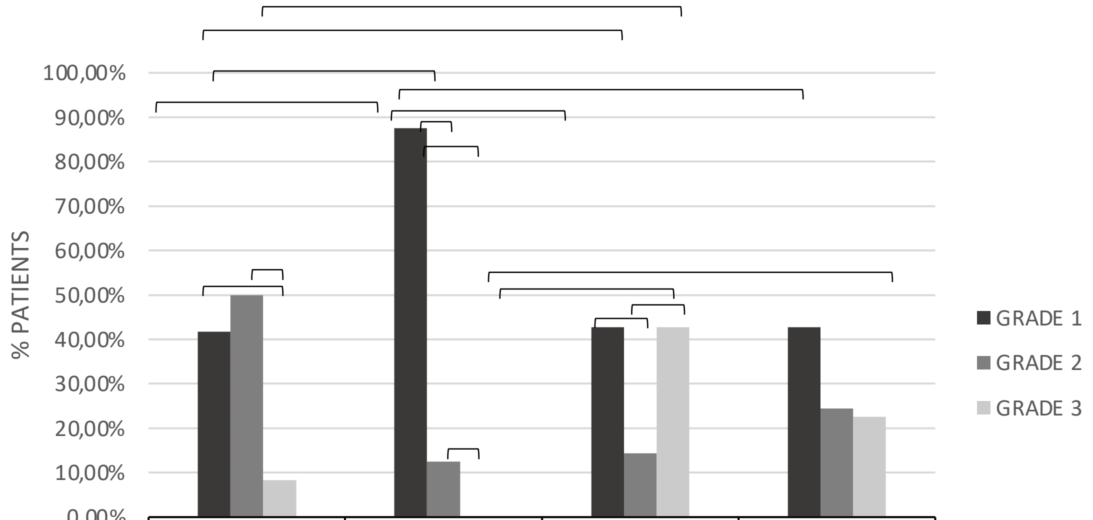

	DN	DP1	DP2	DP
GRADE 1	41,70%	87,50%	42,80%	42,80%
GRADE 2	50%	12,50%	14,30%	24,50%
GRADE 3	8,30%	0	42,80%	22,60%

b)

	ASTHMA						
	P-VALUE ADJUST	DN-DP1	DN-DP2	DN-DP	DP1-DP2	DP1-DP	DP2-DP
GRADE 1	NONE	**$5.6*10^{-5}$**	0.90475	0.90475	**$9.0*10^{-5}$**	**$9.0*10^{-5}$**	1.000
	HOLM	**0.00241**	1.000	1.000	**0.00369**	**0.00369**	1.000
	BONFERRONI	**0.00369**	1.000	1.000	**0.00594**	**0.00594**	1.000
GRADE 2	NONE	**$2.1*10^{-6}$**	**$8.5*10^{-6}$**	0.00313	0.72807	0.04852	0.10152
	HOLM	**0.00010**	**0.00040**	0.09400	1.000	0.87336	1.000
	BONFERRONI	**0.00014**	**0.00056**	0.20680	1.000	1.000	1.000
GRADE 3	NONE	0.00396	**$1.4*10^{-6}$**	0.01010	**$6.1*10^{-11}$**	**$2.0*10^{-6}$**	0.01250
	HOLM	0.11497	**$7.4*10^{-5}$**	0.27260	**$3.7*10^{-9}$**	**$1.0*10^{-4}$**	0.32489
	BONFERRONI	0.26166	**$9.2*10^{-5}$**	0.66637	**$4*10^{-9}$**	**0.00013**	0.82472

	ASTHMA				
	P-VALUE ADJUST	DN	DP1	DP2	DP
G1-2	NONE	0.38608	**$6.4*10^{-4}$**	**0.00016**	0.02570
	HOLM	1.000	**$4.1*10^{-12}$**	**0.00600**	0.56540
	BONFERRONI	1.000	**$4.2*10^{-12}$**	**0.01071**	1.000
G1-3	NONE	**$2.3*10^{-6}$**	**$<2*10^{-16}$**	1.000	0.01250
	HOLM	**0.00011**	**$<2*10^{-16}$**	1.000	0.32489
	BONFERRONI	**0.00015**	**$<2*10^{-16}$**	1.000	0.82472
G2-3	NONE	**$4.7*10^{-8}$**	**0.00041**	**0.00016**	0.78190
	HOLM	**$2.6*10^{-6}$**	**0.01343**	**0.00600**	1.000
	BONFERRONI	**$3.1*10^{-6}$**	**0.02686**	**0.01071**	1.000

Fig. 2 suppl.. Asthma grade distribution for each phenotype considering GEMA classification. a) Percentage of patient in each group suffering asthma. Bar mean statistical difference. P<0.05. b) p-value using different statistical adjust model. When the p-value is lower than 0.05 in the three adjust the sample are considered statistically difference. G1-2,G1-3, G2-3, mean de relationship between both different grade of severity.
